# Inhibitory Effects of Japanese Herbal Medicines Sho-saiko-to and Juzen-taiho-to on Nonalcoholic Steatohepatitis in Mice

**DOI:** 10.1371/journal.pone.0087279

**Published:** 2014-01-22

**Authors:** Yoshihisa Takahashi, Yurie Soejima, Arisa Kumagai, Masato Watanabe, Hiroshi Uozaki, Toshio Fukusato

**Affiliations:** Department of Pathology, Teikyo University School of Medicine, Tokyo, Japan; The Ohio State University, United States of America

## Abstract

Although Japanese herbal medicines (JHMs) are widely used in Japan, only a few studies have investigated their effects on nonalcoholic steatohepatitis (NASH). In the present study, we examined the effect of 4 kinds of JHMs [sho-saiko-to (TJ-9), inchin-ko-to (TJ-135), juzen-taiho-to (TJ-48), and keishi-bukuryo-gan (TJ-25)] on a mouse model of NASH. Db/db mice were divided into 6 groups: control diet (control), methionine- and choline-deficient diet (MCD), and MCD diet supplemented with TJ-9, TJ-135, TJ-48, and TJ-25 (TJ-9, TJ-135, TJ-48, and TJ-25, respectively). All mice were sacrificed after 4 weeks of treatment, and biochemical, pathological, and molecular analyses were performed. Serum alanine aminotransferase levels and liver histology, including necroinflammation and fibrosis, were significantly alleviated in the TJ-9 and TJ-48 groups compared with the MCD group. The expression level of transforming growth factor (TGF)-β1 mRNA in the liver was significantly suppressed by TJ-48. Although the differences were not statistically significant, the expression levels of tumor necrosis factor (TNF)-α and interleukin (IL)-6 were lower, and those of peroxisome proliferators-activated receptor (PPAR)γ were higher in the TJ-9 and/or TJ-48 groups than in the MCD group. Similarly, even though the results were not statistically significant, malondialdehyde levels in liver tissues were lower in the TJ-9 and TJ-48 groups than in the MCD group. We showed that JHMs, especially TJ-9 and TJ-48, inhibited the necroinflammation and fibrosis in the liver of a mouse model of NASH, even though the mechanisms were not fully elucidated. Further studies are needed in the future to investigate the possibility of clinical application of these medicines in the treatment for NASH.

## Introduction

Nonalcoholic fatty liver disease (NAFLD) is a condition in which excessive fat accumulates in the liver of a patient who does not have a history of alcohol abuse. NAFLD is classified into 2 categories: simple steatosis, in which only steatosis is observed, and nonalcoholic steatohepatitis (NASH), in which, in addition to steatosis, lobular inflammation and liver cell injury are observed. NASH is a progressive disease and may develop into liver cirrhosis or hepatocellular carcinoma [1], [2]. NAFLD/NASH, considered as a hepatic manifestation of metabolic syndrome [3], [4], is increasing worldwide along with the increased prevalence of obesity, and at the same time, is the most common chronic liver disease.

The two-hit [5] or multiple-hit [6] hypotheses are generally advocated as the mechanisms underlying the pathogenesis of NAFLD/NASH. It is believed that fat accumulation in the liver is necessary for the occurrence of NAFLD, and further hits, such as oxidative stress, increase of inflammatory cytokines, or decrease of anti-inflammatory cytokines, are necessary for the development of the progressive NASH disease. Animal models are useful for elucidating the pathogenesis and for developing novel treatments for NASH. Although several genetic and nutritional animal models of NAFLD/NASH have been proposed, these models do not replicate the full spectrum of the characteristics of human NAFLD/NASH [7]. Therefore, the combination of 2 or more factors has been occasionally used as a combination model.

Weight loss by modifying the life style is the main treatment for NAFLD. However, a drug therapy for NAFLD needs to be developed because it is often difficult for obese patients to maintain healthy life style changes. Japanese herbal medicines (JHMs) (Kampo medicines) are widely used in Japan, and over 80% of Japanese medical doctors routinely prescribe these medicines [8]. Since JHMs are thought to possess anti-oxidative and anti-inflammatory activities [9]–[11], they might be effective in the treatment for NASH. However, to date, only 2 studies have examined the effects of JHMs on NAFLD (i.e., 1 basic study on sho-saiko-to (TJ-9), keishi-bukuryo-gan (TJ-25), and oren-gedoku-to (TJ-15) [12] and 1 retrospective clinical study on TJ-25 [13]). However, the pathological and molecular analyses of the liver tissues are not complete.

In the present study, to examine the effects of 4 JHMs [TJ-9, inchin-ko-to (TJ-135), juzen-taiho-to (TJ-48), and TJ-25] on NASH, we used a combination model in which db/db mice were fed a methionine- and choline-deficient (MCD) diet. Furthermore, to determine the mechanism underlying the actions of JHMs, we examined the expression levels of various cytokine and receptor genes and the levels of a marker of oxidative stress in the liver. We observed that TJ-9 and TJ-48 inhibited the hepatic lesions of the animal model, although the mechanisms were not fully elucidated.

## Materials and Methods

### Ethics Statement

This study was carried out in strict accordance with the recommendations in the Guide for the Care and Use of Laboratory Animals of the National Institutes of Health. The protocol was approved by the Ethics Committee of Teikyo University (11-015). All the animals received humane care, and all efforts were taken to minimize suffering.

### Medicines

TJ-9, TJ-135, TJ-48, and TJ-25 were kindly donated by Tsumura & Co. (Tokyo, Japan). These medicines contain crude extracts of herbs. The composition of each medicine is summarized in Table 1. The three-dimensional high-performance liquid chromatography (HPLC) profiles of these medicines were also provided from Tsumura & Co. as supplementary data of the products.

**Table 1:** The composition of sho-saiko-to (TJ-9), inchin-ko-to (TJ-135), juzen-taiho-to (TJ-48), and keishi-bukuryo-gan (TJ-25).

	Weight Ratio (g)
TJ-9	
Bupleuri radix	7.0
Pinelliae tuber	5.0
Scutellariae radix	3.0
Zizyphi fructus	3.0
Ginseng radix	3.0
Glycyrrhizae radix	2.0
Zingiberis rhizoma	1.0
TJ-135	
Artemisiae capillari spica	4.0
Gardeniae fructus	3.0
Rhei rhizoma	1.0
TJ-48	
Astragali radix	3.0
Cinnamomi cortex	3.0
Rehmanniae radix	3.0
Paeoniae radix	3.0
Cnidii rhizoma	3.0
Atractylodis lanceae rhizoma	3.0
Angelicae radix	3.0
Ginseng radix	3.0
Hoelen	3.0
Glycyrrhizae radix	1.5
TJ-25	
Cinnamomi cortex	3.0
Paeoniae radix	3.0
Persicae semen	3.0
Hoelen	3.0
Moutan cortex	3.0

### Animals and Experimental Protocols

Thirty-six 8-week-old male BKS.Cg +Lepr^db^/+Lepr^db^/J (db/db) mice were purchased from Charles River Laboratories Japan, Inc. (Yokohama, Japan). After 1 week of acclimation to the environment, mice were divided into 6 groups (n  = 6 for each group): control, MCD, TJ-9, TJ-135, TJ-48, and TJ-25 groups. The mice in the control group were fed a control diet (CRF-1 diet) (Oriental Yeast Co., Tokyo, Japan) ad libitum. The mice in the MCD group were fed an MCD diet (F2MCD) (Oriental Yeast Co.) ad libitum. The mice in the TJ-9, TJ-135, TJ-48, and TJ-25 groups were fed the MCD diet supplemented with 1.5% (w/w) TJ-9, TJ-135, TJ-48, and TJ-25, respectively, ad libitum. Each JHM contained only a small amount of methionine and choline, and the content of methionine and choline in the diet of each JHM group was only about 1% compared with that of the control group [14]-[16]. Thus, it was unlikely that methionine and choline contained in each JHM had major effect on the results of the present study. The diets were stored in a refrigerator at 4°C; the feed containers for mice were refilled with fresh diet 3 times a week, and food consumption was recorded. The mice were maintained in the Laboratory Animal Center of Teikyo University School of Medicine at 25°C and 45% humidity.

The mice were sacrificed by decapitation after 4 weeks (at 13 weeks of age). Mice were starved for 16 h, and body weights were measured before killing. Blood samples of each mouse were collected at decapitation, and the serum was separated by centrifugation. The liver of each mouse was excised and weighed, and samples for histological analysis, RNA purification, and snap freezing were collected.

### Biochemical Analysis of Serum

Serum aspartate aminotransferase (AST), alanine aminotransferase (ALT), total cholesterol (T-Cho), triglyceride (TG), and glucose levels were determined by routine methods using the Hitachi 7180 autoanalyzer (Hitachi High-Technologies Corporation, Tokyo, Japan). Serum insulin, leptin, and adiponectin levels were measured by enzyme-linked immunosorbent assay (ELISA) using the ultrasensitive mouse insulin measuring kit (Morinaga Institute of Biological Science, Inc., Yokohama, Japan), leptin-mouse kit (Shibayagi Co., Ltd, Shibukawa, Japan), and mouse/rat adiponectin ELISA kit (Otsuka Pharmaceutical Co., Ltd., Tokyo, Japan), respectively.

### Histological Analysis

The central parts of the 2 large liver lobes were fixed in 10% formaldehyde solution and routinely processed for light microscopy. Sirius red staining was performed in addition to hematoxylin and eosin staining to assess hepatic fibrosis. The histopathological features of steatohepatitis were evaluated semi-quantitatively, according to the validated histological scoring system by Kleiner et al. [17]. The degree of steatosis was evaluated by the percentage of hepatocytes containing macro- or micro-vesicular fat and graded as follows: grade 0 (<5%), grade 1 (5-33%), grade 2 (>33-66%), and grade 3 (>66%). Lobular inflammation was classified as: 0 (no foci), 1 (<2 foci per 200× field), 2 (2-4 foci per 200× field), or 3 (>4 foci per 200× field). Hepatocellular ballooning was graded as follows: 0 (none), 1 (few balloon cells), or 2 (many cells/prominent ballooning). NAFLD activity score (NAS) was calculated as the sum of the scores of steatosis (0-3), lobular inflammation (0-3), or hepatocellular ballooning (0-2). Fibrosis staging was classified as follows: 0 (none), 1 (perisinusoidal or periportal), 2 (perisinusoidal and portal/periportal), 3 (bridging fibrosis), or 4 (cirrhosis).

### Immunohistochemical Staining and Morphometric Image Analysis

Immunohistochemical staining for α-smooth muscle actin (SMA) was performed to detect activated hepatic stellate cells (HSCs). The paraffin sections of liver specimens were immunostained with anti-SMA monoclonal antibody (clone: 1A4, dilution: 1:100, DakoCytomation, Glostrup, Denmark) using the EnVision™ FLEX system (DakoCytomation). Antigen retrieval was performed by heating for 20 min in citrate buffer at pH 6.0 in a water bath.

Image analysis was performed to objectively evaluate the degree of fibrosis and the number of activated HSCs. Photomicrographs of 3 randomly selected intralobular 400× fields were taken for each Sirius red and SMA staining slide. The frequencies of Sirius red- or SMA-positive areas in those photomicrographs were analyzed with the image analysis software WinROOF (Mitani Corporation, Fukui, Japan).

### Real-time Reverse Transcription Polymerase Chain Reaction (RT-PCR)

Total RNA was isolated from the liver using the AllPrep DNA/RNA/Protein Mini kit (Qiagen, Valencia, CA, USA) and reverse transcribed using the QuantiTect Reverse Transcription kit (Qiagen). The quality and quantity of the RNA samples were determined using the Agilent 2100 Bioanalyzer (Agilent Technologies, Waldbronn, Germany). Quantitative real-time PCR was performed using ABI 7300 real-time PCR system and the Power SYBR Green PCR Master Mix kit (Life Technologies, Carlsbad, CA, USA). The primers for complementary DNA amplification of tumor necrosis factor (TNF)-α, interleukin (IL)-6, peroxisome proliferators-activated receptor (PPAR)α, PPARγ, transforming growth factor (TGF)-β1, and β-actin genes are listed in Table 2. β-Actin was used as internal control. All the samples were assayed in triplicate. Absolute quantification of the copy number of each gene was performed using a standard curve constructed with serially diluted control plasmids obtained by TA cloning from the PCR products of the liver tissues of mice in the control group. The mRNA expression level of each gene was normalized by the expression level of β-actin mRNA.

**Table 2:** Primers used in real-time reverse transcription polymerase chain reaction.

Genes	Forward primers (5′→3′)	Reverse primers (5′→3′)
TNF-α	GGTGATCGGTCCCCAAAGGGATG	TGGGCTACAGGCTTGTCACTCGAA
IL-6	AGACAAAGCCAGAGTCCTTCAGAGA	GCCACTCCTTCTGTGACTCCAGC
PPARα	TGTGGGGACAAGGCCTCAGGGT	TGCAGCTCCGATCACACTTGTCGT
PPARγ	TCAGAAGTGCCTTGCTGTGGGGA	GAGATCTCCGCCAACAGCTTCTCCT
TGF-β1	GGCACCGGAGAGCCCTGGATA	AATGTACAGCTGCCGCACACAGC
β-actin	TTCGTTGCCGGTCCACACCC	TTTGCACATGCCGGAGCCGT

IL, interleukin; PPAR, peroxisome proliferators-activated receptor; TGF, transforming growth factor; TNF, tumor necrosis factor.

### Malondialdehyde (MDA) Levels in the Liver

To assess the oxidative injury of lipids in the liver, hepatic MDA level was measured using the MDA assay kit (Japan Institute for the Control of Aging, Fukuroi, Japan), according to the manufacturer’s instructions.

### Statistical Analysis

Data were indicated as mean ± standard deviation. One-way analysis of variance (ANOVA) was performed to assess the significance of the differences. P<0.05 was considered statistically significant.

## Results

### General Observations

No mice died during the experiment. Although food consumption was significantly lower in the MCD group than in the control group, JHM supplementation did not influence food consumption (data not shown). In addition, while liver weight, body weight, and liver/body weight ratio were significantly lower in the MCD group than in the control group, JHM supplementation did not influence these weights (data not shown).

### Biochemical Data of Serum

Table 3 shows the serum data corresponding to each group. AST levels were significantly higher in the MCD group than in the control group; they were lower in the TJ-9, TJ-48, and TJ-25 groups (especially in the TJ-9 group) than in the MCD group, but the differences were not statistically significant. ALT levels were significantly higher in the MCD group than in the control group, and they were significantly lower in the TJ-9 and TJ-48 groups than in the MCD group. T-Cho levels were significantly higher in the MCD group than in the control group, and they were significantly lower in the TJ-9 group than in the MCD group. TG levels were significantly lower in the MCD group than in the control group, and significantly further lower in the TJ-135 group than in the MCD group. Although glucose and insulin levels were significantly lower in the MCD group than in the control group, JHM supplementation did not significantly influence these levels. Leptin levels were significantly higher in the MCD group than in the control group and significantly lower in the TJ-135 and TJ-25 groups than in the MCD group. Adiponectin levels were significantly higher in the MCD group than in the control group, and significantly lower in the TJ-135 group than in the MCD group.

**Table 3:** Biochemical data of serum in experimental groups.

	control	MCD	TJ-9	TJ-135	TJ-48	TJ-25
AST (IU/L)	136.7±12.1	240.0±54.8^a^	173.3±34.4	281.7±140.2	223.3±37.2	225.0±37.8
ALT (IU/L)	81.7±13.3	123.3±41.8^a^	72.0±8.4^b^	121.7±30.6	78.3±17.2^b^	95.0±28.8
T-Cho (mg/dL)	171.7±18.3	241.7±35.4^a^	195.0±18.7^b^	232.0±8.4	215.0±21.7	238.3±44.9
TG (mg/dL)	138.0±13.0	66.7±10.3^a^	61.7±13.3	46.7±8.2^b^	60.0±12.6	55.0±5.5
Glucose (mg/dL)	736.7±112.9	101.7±34.9^a^	95.0±32.7	140.0±33.5	158.0±23.9	130.0±32.9
Insulin (ng/mL)	2.84±1.13	1.59±0.26^a^	1.09±0.74	1.76±0.94	1.29±0.32	1.13±0.60
Leptin (ng/mL)	7.36±0.79	9.99±1.91^a^	9.17±2.35	6.99±0.92^b^	8.01±0.81	7.26±0.30^b^
Adiponectin (μg/mL)	23.4±3.0	40.7±6.1^a^	44.1±4.3	33.0±1.3^b^	35.0±5.9	34.1±7.0

Data are presented as mean ± standard deviation.

^a^ Significantly different from the control group (P<0.05). ^b^Significantly different from the MCD group (P<0.05).

ALT, alanine aminotransferase; AST, aspartate aminotransferase; T-Cho, total cholesterol; TG, triglyceride.

### Histological Findings

**Figure 1:**
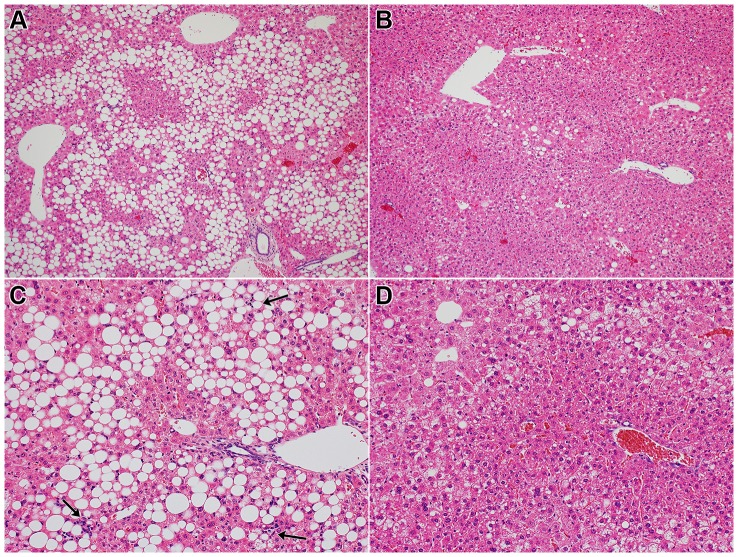
Photomicrographs of the liver. Mice of the MCD group show marked hepatic steatosis (A), but steatosis is suppressed in mice of the TJ-48 group (B). Necroinflammatory foci (arrows) are scattered in the liver of mice of the MCD group (C), but lobular inflammation is suppressed in mice of the TJ-9 group (D). (Hematoxylin and eosin stain; A and B: ×100; C and D: ×200).

The mice in the MCD group showed liver histopathology consistent with NASH. The localization of steatosis was predominant in zone 3 or azonal/panacinar, while that of fibrosis was predominant in zone 3. Thus, the histological pattern of the present animal model resembled that of human NASH. Table 4 summarizes the histological findings of each group. Steatosis grade was especially lower in the TJ-9 and TJ-48 groups than in the MCD group, and the difference was statistically significant for the TJ-48 group (Figure 1A and B). Lobular inflammation was more severe in the MCD group than in the control group; it was especially milder in the TJ-9 and TJ-48 groups than in the MCD group, and the difference was statistically significant for the TJ-9 group (Figure 1C and D). Ballooning degeneration was significantly more severe in the MCD group than in the control group, and significantly milder in the TJ-9, TJ-135, and TJ-48 groups than in the MCD group. NAS was significantly higher in the MCD group than in the control group, and it was significantly lower in the TJ-9 and TJ-48 groups than in the MCD group. Fibrosis stage was higher in the MCD group than in the control group; it was especially lower in the TJ-9 and TJ-48 groups than in the MCD group, and the difference was statistically significant for the TJ-48 group.

**Table 4:** Histological findings in experimental groups.

	control	MCD	TJ-9	TJ-135	TJ-48	TJ-25
Steatosis grade	2.33±0.52	1.83±0.75	1.33±0.52	2.00±0.00	1.00±0.63^b^	1.67±0.52
Lobular inflammation	1.00±0.00	1.50±0.55	0.67±0.52^b^	1.67±0.52	0.83±0.41	1.17±0.75
Ballooning	0.00±0.00	1.67±0.52^a^	0.33±0.52^b^	1.00±0.00^b^	0.67±0.52^b^	1.17±0.75
NAS	3.33±0.52	5.00±1.41^a^	2.33±1.03^b^	4.67±0.52	2.50±1.38^b^	4.00±1.90
Fibrosis stage	0.17±0.41	0.67±0.52	0.17±0.41	0.50±0.55	0.00±0.00^b^	0.33±0.52

Data are presented as mean ± standard deviation.

^a^ Significantly different from the control group (P<0.05).

^b^ Significantly different from the MCD group (P<0.05).

NAS, NAFLD activity score.

### Immunohistochemical Staining and Morphometric Analysis of Fibrosis and Activated HSCs

**Figure 2:**
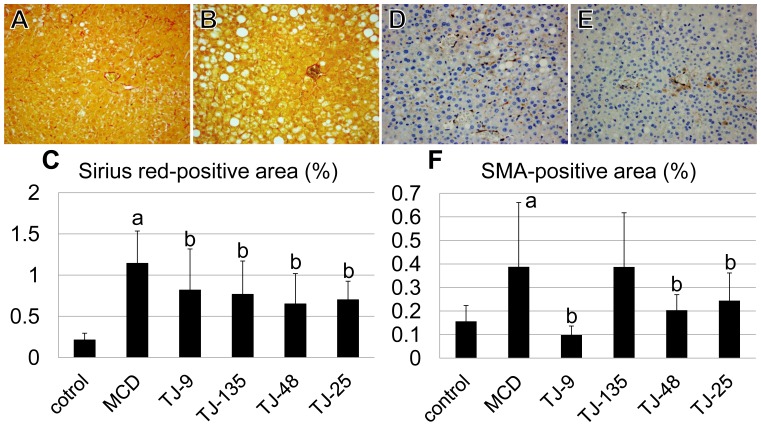
The frequency of Sirius red-positive and SMA-positive area. (A-C) The frequency of Sirius red-positive area is significantly higher in the MCD group than in the control group, and significantly lower in all the JHM groups than in the MCD group. The Sirius red-positive area is very small in the control group. (A: photomicrograph of a mouse of the MCD group, B: photomicrograph of a mouse of the TJ-48 group, ×400) (D–F) The rate of the SMA-positive area is significantly higher in the MCD group than in the control group, and significantly lower in the TJ-9, TJ-48, and TJ-25 groups than in the MCD group. (D: photomicrograph of a mouse of the MCD group, E: photomicrograph of a mouse of the TJ-9 group, ×400). ^a^Significantly different from the control group (P<0.05). ^b^Significantly different from the MCD group (P<0.05).

The frequency of the Sirius red-positive area, reflecting the degree of fibrosis, in the hepatic lobules was significantly higher in the MCD group than in the control group, and significantly lower in all JHM groups (especially in the TJ-48 group) than in the MCD group (Figure 2A-C). The mice in the control group showed very slight perisinusoidal Sirius red-positivity. The rate of SMA-positive area, reflecting the number of activated HSCs, in the hepatic lobules was significantly higher in the MCD group than in the control group, and significantly lower in the TJ-9, TJ-48, and TJ-25 groups (especially in the TJ-9 group) than in the MCD group (Figure 2D–F).

### Gene Expression in the Liver

**Figure 3:**
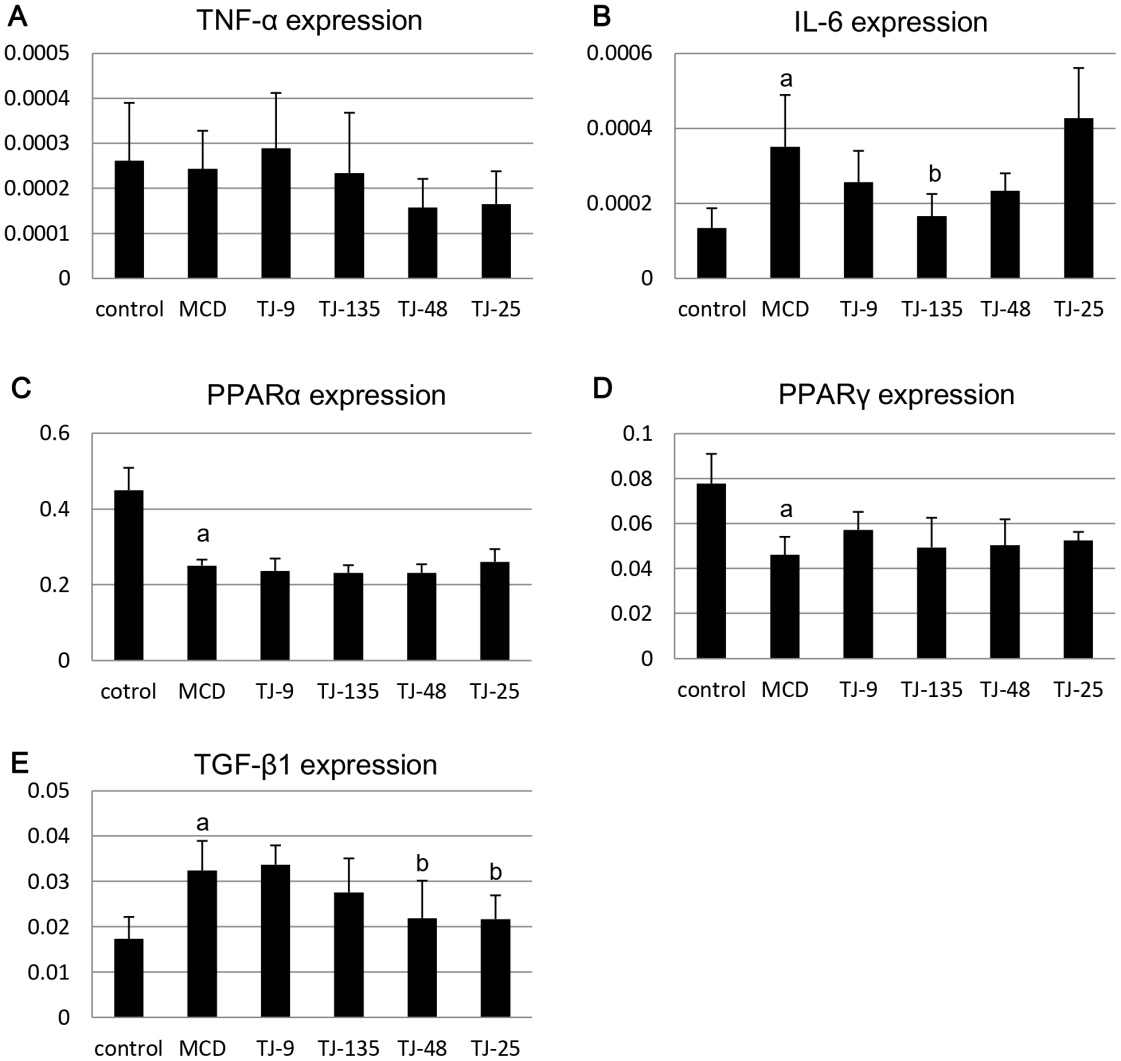
Expression levels of cytokine and receptor genes in the liver. TNF-α expression levels are lower in the TJ-48 and TJ-25 groups than in the MCD group (A). IL-6 expression levels are significantly higher in the MCD group than in the control group; they are lower in the TJ-9, TJ-135, and TJ-48 groups than in the MCD group, and the difference is statistically significant for the TJ-135 group (B). Although PPARα expression levels are significantly lower in the MCD group than in the control group, they are not influenced by JHM supplementation (C). PPARγ expression levels are significantly lower in the MCD group than in the control group; they are higher in all the JHM groups (especially in the TJ-9 group) than in the MCD group (D). TGF-β1 expression levels are significantly higher in the MCD group than in the control group, and significantly lower in the TJ-48 and TJ-25 groups than in the MCD group (E). ^a^Significantly different from the control group (P<0.05). ^b^Significantly different from the MCD group (P<0.05).

Figure 3 shows the expression levels of TNF-α, IL-6, PPARα, PPARγ, and TGF-β1 mRNA in the liver determined by real-time RT-PCR. TNF-α expression levels were lower in the TJ-48 and TJ-25 groups than in the MCD group, but the differences were not statistically significant. IL-6 expression levels were significantly higher in the MCD group than in the control group; they were lower in the TJ-9, TJ-135, and TJ-48 groups than in the MCD group, and the difference was statistically significant for the TJ-135 group. Although PPARα expression levels were significantly lower in the MCD group than in the control group, they were not influenced by JHM supplementation. PPARγ expression levels were significantly lower in the MCD group than in the control group; they were higher in all the JHM groups (especially in the TJ-9 group) than in the MCD group, but the differences were not statistically significant. TGF-β1 expression levels were significantly higher in the MCD group than in the control group, and they were significantly lower in the TJ-48 and TJ-25 groups than in the MCD group.

### MDA Levels in the Liver

**Figure 4:**
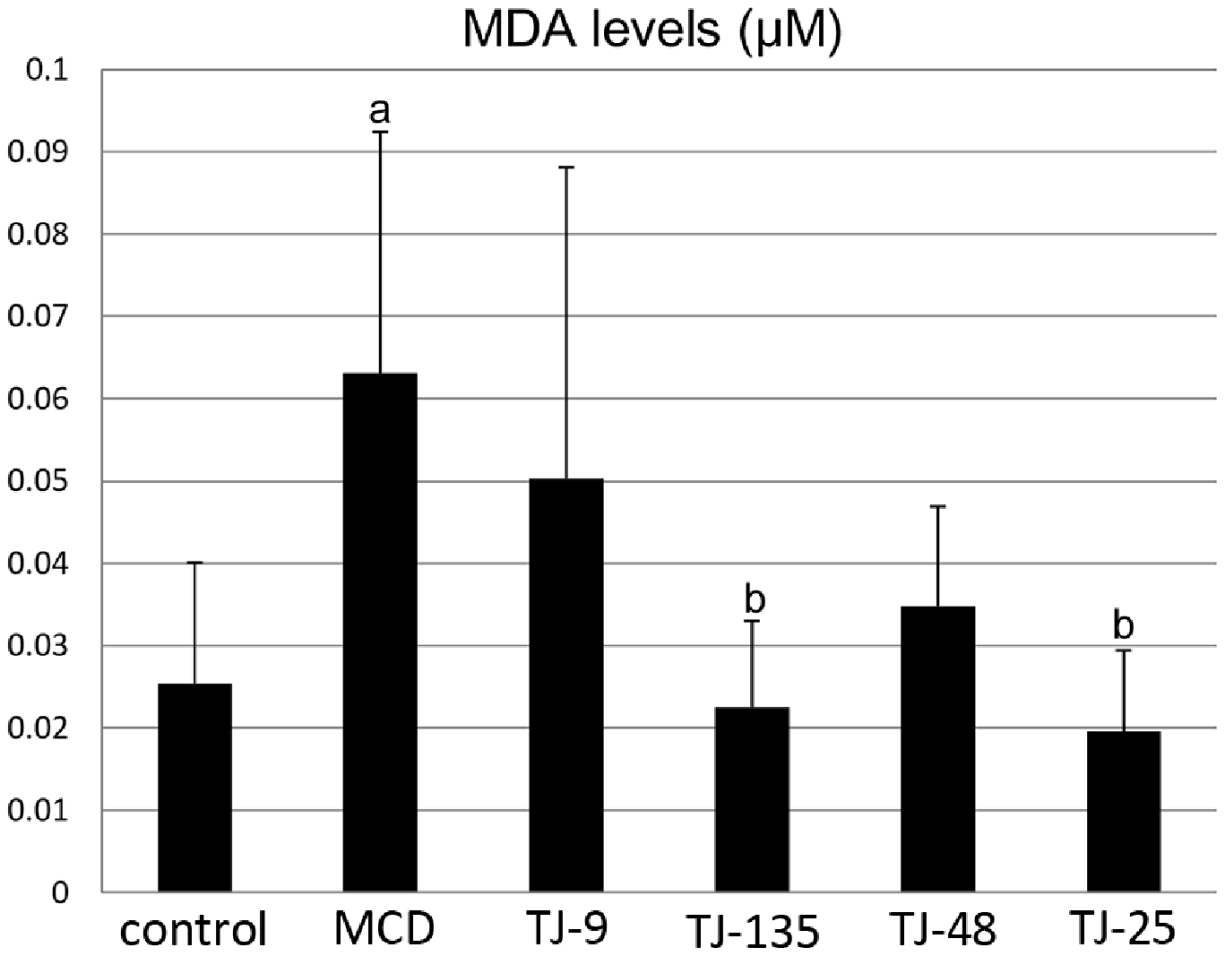
MDA levels in the liver. MDA levels are significantly higher in the MCD group than in the control group; they are lower in all the JHM groups than in the MCD group, and the differences are statistically significant for the TJ-135 and TJ-25 groups. ^a^Significantly different from the control group (P<0.05). ^b^Significantly different from the MCD group (P<0.05).

Figure 4 shows the MDA levels in the liver of each group. MDA levels were significantly higher in the MCD group than in the control group; they were lower in all the JHM groups than in the MCD group, and the differences were statistically significant for the TJ-135 and TJ-25 groups.

## Discussion

This study shows that JHMs (especially TJ-9 and TJ-48) significantly improved the serum ALT levels and liver histology, including the degree of fibrosis, in an animal model of NASH. Based on our molecular analysis, TJ-48 significantly suppressed the expression level of TGF-β1 mRNA in the liver. This is the first study showing that JHMs significantly improve serum aminotransferase levels and liver histology in animal models of NAFLD/NASH. Additionally, this study investigated for the first time the effects of JHMs on the expression levels of cytokine and receptor genes in livers affected by NASH. Furthermore, this is the first study to show the beneficial effects of TJ-48 on NAFLD/NASH.

Recently, scientific research on Japanese traditional medicines has been progressing, and several rigorous clinical and basic research studies have confirmed the effects of these medicines [18]. More than 100 kinds of JHMs have been developed to date. In the present study, we examined 4 medicines (TJ-9, TJ-135, TJ-48, and TJ-25) because we speculated that these medicines might be the most promising for the treatment for NASH based on previous studies. TJ-9 has been confirmed to possess anti-oxidative, anti-inflammatory, anti-fibrogenic, and anti-tumor effects in the liver in previous animal experiments [10], [19]. TJ-135 has been confirmed to possess anti-inflammatory and anti-fibrogenic effects in the liver in previous animal experiments [20], [21]. TJ-48 was confirmed to possess anti-oxidative, anti-inflammatory, and anti-tumor effects in the liver in a previous animal experiment [22]. There have been only 1 basic study [12] and 1 retrospective clinical study [13] on the effects of JHMs on NAFLD, and TJ-25 showed positive effects in both those studies.

Db/db mice, possessing a natural mutation in the leptin receptor (*Ob-Rb*) gene [23], are widely used as model mice of NAFLD and obesity/diabetes; however, these mice do not spontaneously develop steatohepatitis or liver fibrosis. Accordingly, in the present study, we used the combination model of db/db mice and MCD diet. The MCD diet lacks methionine and choline, which are essential for hepatic β-oxidation and production of very low-density lipoprotein [24]. Furthermore, this diet induces oxidative stress [25], [26] and changes in cytokine and adipocytokine levels [27], contributing to the liver injury. Although the MCD model causes severe hepatic inflammation, oxidative stress, and fibrogenesis, its metabolic profile is opposite to that of the typical human NASH [7].

In the present study, TJ-9 and/or TJ-48 administration caused significant reduction in serum ALT levels, and furthermore, showed significant inhibitory effects on steatosis, lobular inflammation, ballooning, NAS, and fibrosis stage on histological analysis. The inhibitory effect of these JHMs on fibrosis was further confirmed by image analysis of Sirius red staining. In general, Sirius red staining shows patches of linear perisinusoidal staining in old mice but not in young mice [28]. The mice used in the present study were young, with those in the control group showing very slight perisinusoidal Sirius red-positivity. HSCs play a pivotal role in inducing hepatic fibrosis [29]. Although quiescent HSCs cannot be observed by usual staining, activated HSCs are immunohistochemically positive for SMA. The results of image analysis for SMA staining suggested that these JHMs decreased the number of activated HSCs. These inhibitory effects on hepatic fibrosis are important because advanced fibrosis is closely associated with liver-related mortality in NASH [30].

Moreover, the present study analyzed the expression levels of cytokine and receptor genes in the liver. In the present study, although the differences were not statistically significant, the expression levels of TNF-α (a representative pro-inflammatory cytokine) were decreased by TJ-48 and TJ-25. In addition, the expression levels of IL-6 were decreased by TJ-9, TJ-135, and TJ-48. IL-6 is hypothesized to sensitize the liver to injury, stimulate hepatocyte apoptosis, induce insulin resistance, and participate in NASH development [31]. In the present study, although the differences were not statistically significant, the expression levels of PPARγ were increased by all the JHMs examined (especially by TJ-9). It has been reported that PPARγ activation increases insulin sensitivity in peripheral tissues, increases adiponectin levels, and inhibits the activation and proliferation of HSCs [32]. In addition, in accordance with the amelioration of the hepatic fibrosis, TJ-48 and TJ-25 significantly decreased the expression levels of TGF-β1. It is known that TGF-β1 plays a pivotal role in hepatic fibrosis by mediating the activation of HSCs and their production of extracellular matrix proteins [33], [34]. However, the molecular mechanisms for the inhibitory effects of TJ-9 and TJ-48 on this animal model of NASH have not been fully elucidated in the present study, because, among the five genes tested, only TGF-β1 expression was significantly affected by TJ-9 or TJ-48.

MDA is a widely used marker for oxidative stress. In the present study, all the JHMs examined decreased the MDA levels in the liver. However, the suppression of MDA was more prominent in TJ-135 and TJ-25 than in TJ-9 and TJ-48. Considering that TJ-9 and TJ-48 showed more prominent inhibitory effects on NASH serologically and histologically than TJ-135 and TJ-25, it was difficult to explain the inhibitory effects of JHMs on NASH only by their anti-oxidative activity.

There has been only 1 report on the effects of JHMs on animal models of NAFLD in which a high-cholesterol diet-fed rabbit model was studied [12]. Although TJ-25 improved lipid profiles and oxidative stress, no JHMs examined significantly improved the plasma AST or ALT levels. Since the hepatic inflammation in the high-cholesterol diet model is milder than that in the MCD diet model [7], this may be the cause of the above-mentioned results in the previous study. Neither liver histopathology nor expression levels of cytokine and receptor genes were analyzed in the previous study. Thus, the present study showed for the first time that in animal models of NAFLD/NASH, JHMs improve serum aminotransferase levels, alleviate the liver histology, and regulate the expression levels of cytokine genes.

However, the present study has a limitation. Because the metabolic status of the MCD model is different from that of human NASH, the significance of the changes of serum T-Cho, TG, leptin, and adiponectin levels upon JHM administration in the present study is unclear. In addition, the present study did not elucidate which components of TJ-9 and TJ-48 were effective in inhibiting NASH. JHMs contain numerous chemicals, but the accurate composition has not been fully elucidated. In the present study, TJ-9 showed clear inhibitory effects on NASH. Among the chemical components of TJ-9, whose three-dimensional HPLC profile is displayed in Figure S1, baicalin was reported to ameliorate metabolic disorders and hepatic steatosis in rats given a high-fat diet [35]. Furthermore, baicalein was reported to stimulate nuclear respiratory factor 2-mediated antioxidant response element transactivation [36]. However, Scutellariae radix, the major source of baicalin and baicalein, is not contained in TJ-48, and further studies are necessary to elucidate the active components of TJ-9 and TJ-48 in the inhibition of NASH.

Based on the present results, it is expected that JHMs, especially TJ-9 and TJ-48, will be clinically applied in the treatment for NASH in the future. However, several points should be further elucidated before the commencement of clinical studies. First, the intake of JHMs per body weight in the present study was more than 10 times higher than the usual dosage in humans. Although differences between species exist, the dose-response relationship should be examined. Although no obvious side effects were observed in the present study, JHMs are known to possess side effects. Especially, usage of TJ-9 for liver cirrhosis or liver cancer patients is now considered as a contraindication because interstitial pneumonia may occur [37], [38]. A detailed examination of the side effects is needed in the future. Furthermore, whether these JHMs exert inhibitory effects on other animal models of NASH should be examined. In this regard, we have confirmed that TJ-9, TJ-135, and TJ-48 also inhibit high-fat diet-induced NASH in db/db mice, a model that reflects the metabolic status of NASH more correctly than the MCD diet model does (unpublished data).

In summary, we showed that JHMs, especially TJ-9 and TJ-48, inhibited necroinflammation and fibrosis in the liver of an animal model of NASH. Furthermore, we found that TJ-48 significantly suppressed the expression level of TGF-β1 in the liver. Further studies are needed in the future to elucidate the possibility of clinical applications of these JHMs in the treatment for NASH.

## Supporting Information

Figure S1 **Three-dimensional HPLC profile of TJ-9.** TJ-9 contains many chemical components including baicalin and baicalein (data provided by Tsumura & Co. as supplementary data of the product).
(TIF)
