# Supplementary figures and images for: Inhibitory Effects of Japanese Herbal Medicines Sho-saiko-to and Juzen-taiho-to on Nonalcoholic Steatohepatitis in Mice
Source: PLoS One. 2014 Jan 22;9(1):e87279. doi: 10.1371/journal.pone.0087279 (PMC3899375; doi:10.1371/journal.pone.0087279)

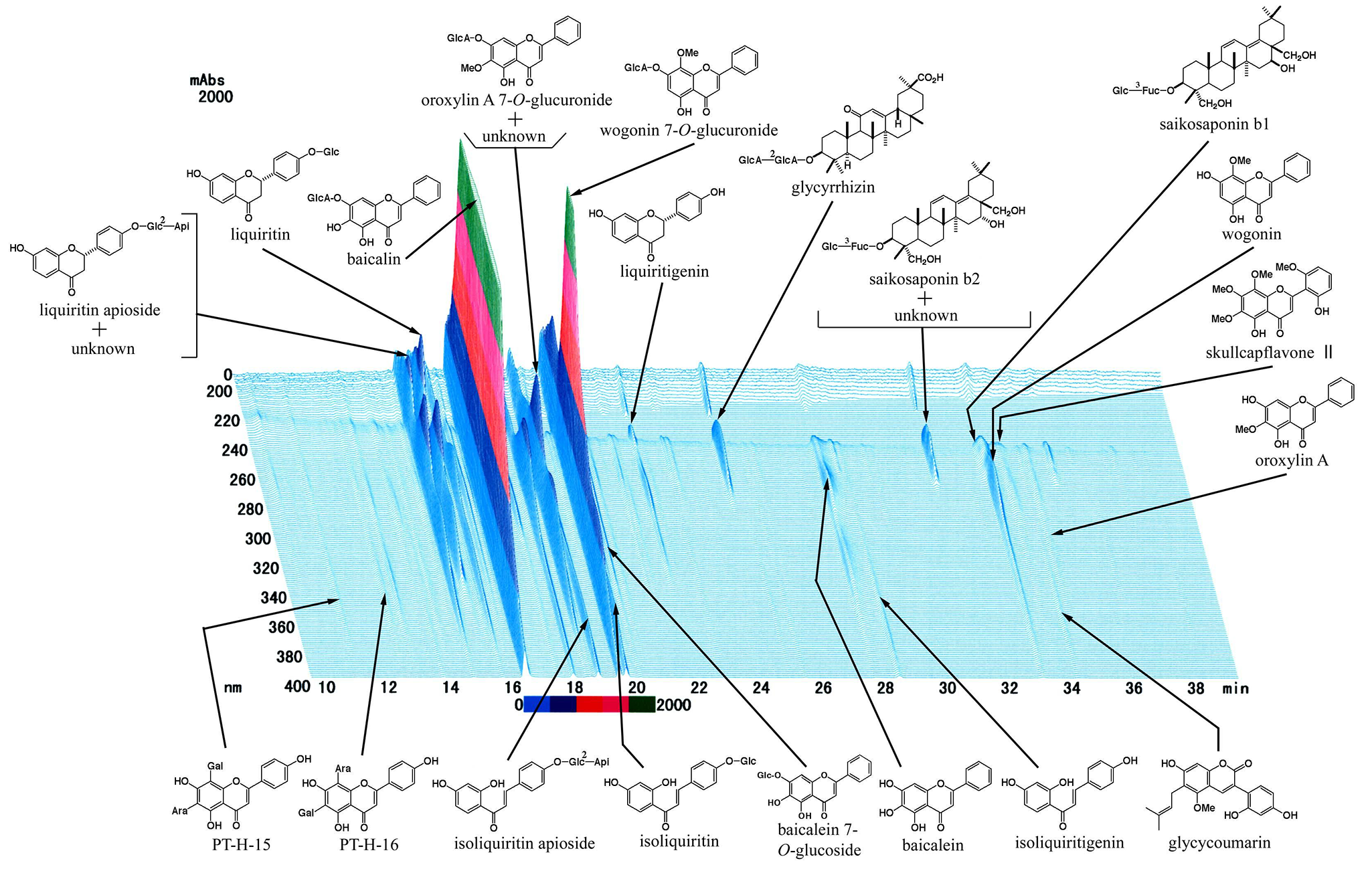

Supplement: Figure S1 — Three-dimensional HPLC profile of TJ-9. TJ-9 contains many chemical components including baicalin and baicalein (data provided by Tsumura & Co. as supplementary data of the product). (TIF) [file pone.0087279.s001.tif]
